# Bone Disease in Long-Term Lung Transplant Survivors

**DOI:** 10.3390/life13040928

**Published:** 2023-04-01

**Authors:** Giorgia Grassi, Elisa Cairoli, Lucrezia Maria Silvana Gentile, Iacopo Chiodini, Marta Zampogna, Alberto Ghielmetti, Letizia Corinna Morlacchi, Valeria Rossetti, Lorenzo Rosso, Ilaria Righi, Mario Nosotti, Maura Arosio, Francesco Blasi, Cristina Eller Vainicher

**Affiliations:** 1 Unit of Endocrinology, Fondazione IRCCS Cà Granda Ospedale Maggiore Policlinico, 20122 Milan, Italy; 2 Department of Clinical Sciences and Community Health, University of Milan, 20123 Milan, Italy; 3 Unit for Bone Metabolism Diseases and Diabetes, Lab of Endocrine and Metabolic Research, Istituto Auxologico Italiano, IRCCS, 20122 Milan, Italy; 4 Unit of Endocrinology, Ospedale Niguarda Cà Granda, 20162 Milan, Italy; 5 Department of Medical Biotechnology and Translational Medicine, University of Milan, 20123 Milan, Italy; 6 Respiratory Unit, Cystic Fibrosis Adult Center, Fondazione IRCCS Ca’ Granda Ospedale Maggiore Policlinico, 20122 Milan, Italy; 7 Department of Pathophysiology and Transplantation, University of Milan, 20123 Milan, Italy; 8 Thoracic Surgery and Lung Transplant Unit, Fondazione IRCCS Ca’ Granda Ospedale Maggiore Policlinico, 20122 Milan, Italy

**Keywords:** lung transplantation, trabecular bone score, vertebral fractures, cystic fibrosis

## Abstract

Background: During the first two years after lung transplantation (LTx), the incidence of fragility fractures (FX) is estimated to be 15–50% and it is lower in patients with cystic fibrosis (CF) as compared with other end-stage lung diseases (nCF). The aim of our study is to compare the skeletal outcomes, after the first 2 years post-LTx, in long-term survivors with CF and nCF. Materials and Methods: We evaluated the FX rate, the changes in bone mineral density (BMD) and trabecular bone score (TBS) in 68 patients (38 CF and 30 nCF) who underwent LTx in our center and with a follow-up after LTx longer than 5 years (7.3 ± 2.0 years). Results: After the second year post-LTx: (i) the FX rate was lower than during the first two years post-LTx (4.4 vs. 20.6%, *p* = 0.004), with no difference between CF and nCF patients (5.3 vs. 3.3%, *p* = 0.589); (ii) BMD at lumbar spine, femoral neck and total hip remained stable (−1.6 ± 1.0 vs. −1.4 ± 1.1, *p* = 0.431, −1.8 ± 0.9 vs. −1.9 ± 0.9, *p* = 0.683, −1.5 ± 0.9 vs. −1.4 ± 0.9, *p* = 0.678, respectively) as well as TBS (1.200 ± 0.124 vs. 1.199 ± 0.205, *p* = 0.166). Conclusions: After the second year post-LTx, the skeletal complications become less frequent and have similar incidence in patients with CF and nCF.

## 1. Introduction

End-stage respiratory failure is associated with a deterioration in bone health and lung transplant (LTx) candidates often present osteoporosis and fragility fractures (FX), with an estimated prevalence reaching 60% and 50%, respectively [1,2,3,4,5,6,7,8,9,10,11,12]. The immediate post-LTx is burdened by a significant bone mass density (BMD) reduction and a relevant incidence of FX as high as 50% when also morphometric vertebral fractures are considered [13,14,15,16,17,18].

The initial worsening in bone status after LTx is partly explained by immobilization and use of immunosuppressive therapies (especially high-dose glucocorticoids, GC), even though, also, the LTx per se seems to play a role [16,17]. Although there are only a few randomized placebo-controlled trials in the literature exploring the role of bisphosphonates (neridronate and pamidronate) in this setting, available data support their use to prevent the BMD loss in the peri-transplant period. However, scarce data are available on their antifracture effect and the factors predictive of FX after LTx are largely unknown [13,19,20]. In a previous study, we investigated the role of the underlying lung disease on bone health both before and during the first two years after LTx in our cohort of patients [12]. Our data suggested that, in spite of a more severe skeletal impairment before LTx, the early post-LTx clinical course of patients with CF was more favorable, as incident FX—mainly clinical vertebral FX—occurred almost entirely in patients affected with other end-stage lung disease (nCF). Additionally, bone microarchitecture—indirectly estimated by trabecular bone score (TBS)—improved significantly only in CF patients [12]. It should be noted that both groups were treated with bisphosphonates in the peri-transplant period (73% of CF and 79% of nCF patients introduced pharmacological therapy within 6 months after LTx) regardless of BMD and prevalent FX [12]. The incidence of FX in the early post-LTx was strongly associated with nCF lung disease and, in this subgroup of patients, with the presence of osteoporosis at the time of LTx [12].

The few studies in the literature on long-term skeletal outcomes in LTx recipients suggest that, over time, BMD becomes stable and the FX rate decreases [15,21,22] but no data are available on possible differences between CF and nCF patients.

The aim of our study is to compare the skeletal outcomes after the first 2 years post-LTx between long-term LTX survivors with CF and those with nCF.

## 2. Materials and Methods

### 2.1. Patients

In this retrospective longitudinal study, we included all consecutive patients aged ≥20 years with end-stage lung disease referred as LTx candidates to the Fondazione IRCCS Cà Granda Ospedale Maggiore Policlinico (Milan, Italy) from January 2009 to December 2016 who eventually underwent LTX and survived >5 years thereafter. The exclusion criteria were other organ transplantation before LTx or within 5 years after and incomplete bone workup at enrollment.

During the study period, 136 patients underwent LTx. Among these patients, 16 patients did not complete the bone workup at enrollment, 8 underwent other solid organ transplant, 43 patients died within 5 years after LTx and 1 patient was lost at follow-up. Among the remaining 68 patients included in this study, 38 and 30 were CF and nCF, respectively. In the nCF group 5, 12, 5 and 8 patients were affected with chronic-obstructive pulmonary disease (COPD), idiopathic pulmonary fibrosis (IPF), connective tissue disease associated with interstitial lung disease (CTD-ILD) and rare lung diseases (RLD), respectively.

The steroid regimen used at our center is methylprednisolone intraoperatively 500–1000 mg (intravenous—IV—weight-adjusted dose) at time of reperfusion, 0.5 mg/Kg IV twice per day for the first 3 days postoperatively, then oral prednisone tapered over 18–24 months post-transplant (discharge–3 months 20 mg, 3–6 months 15 mg, 6–12 months 10 mg, 12–18 months 7.5 mg, and >18 months 5 mg).

A bone-active therapy was proposed to all patients during the workup before being considered admissible on the transplant list regardless of BMD and prevalent FX [12]. The type of drug was chosen according to the reimbursement criteria released by the Italian Drug Agency (https://www.aifa.gov.it/nota-79, accessed on 6 February 2023). From the third year post-LTX, the therapy discharge was tailored according to the fracture risk, which was reassessed at each medical check-up.

A cholecalciferol supplementation per os was introduced in all patients. The standard maintenance cholecalciferol dose was 400 or 800 IU daily plus 100,000 IU bolus per month, in addition to specific multivitamin supplementation in CF group [23]. Calcium supplementation was started only in subjects with a low dietary calcium intake in order to meet the recommended daily demand [24] and hormonal replacement therapy was introduced in patients with hypogonadism in the absence of contraindications.

### 2.2. Methods

We collected information about body mass index (BMI), daily calcium intake, current and past exposure to GC, hypogonadism, previous and ongoing bone-active therapies and prevalent FX during the workup before the patient was considered admissible on the transplant list. The calcium intake was assessed using a validated 7-day food frequency questionnaire [25]. A fracture was considered prevalent and due to bone fragility if it had occurred before our evaluation and without any evident trauma or after a low-energy trauma (e.g., a fall from a standing height) and was confirmed by radiological reports [26]. We recorded the time spent on the waiting list before LTx and the rejection episodes during the follow-up. Data were confirmed by reviewing the medical records.

The 25-hydroxyvitamin-D (25OHD) and testosterone levels were measured by chemiluminescent (reference interval = 75–250 nmol/L, 30–100 ng/mL) and electrochemiluminescent immunoassay (reference interval = males 80–242 nmol/L, 2.8–8.4 ng/mL), respectively, during the workup before the patient was considered admissible on the transplant list and at each bone follow-up (approximately every 2 years). Serum samples were collected and stored at −20 °C until assayed, regardless of the season. Hypovitaminosis-D was defined as 25OHD below the reference range (75 nmol/L, 30 ng/mL) and severe deficiency as 25OHD below 25 nmol/L (10 ng/mL). Untreated hypogonadism was defined as the association of low testosterone levels and signs and symptoms of hypogonadism in males and secondary amenorrhea (lasting longer than 3-month) in females, without ongoing hormonal replacement therapy.

We also collected dual X-ray absorptiometry (DXA) and spine radiograph data during the workup before the patient was considered admissible on the transplant list 2 years after LTx and at last available follow-up. The DXA (Hologic Discovery, Software version 13.3:3, Bedford MA, USA) scan was carried out to measure BMD at lumbar spine (LS; in vivo precision 1.0%), total hip (TH; in vivo precision 1.7%), femoral neck (FN; in vivo precision 1.8%) and TBS. The BMD data were expressed as T-score (BMD difference in standard deviation (SD) in comparison with healthy 30-year-old individuals of the same gender, as provided by the manufacturer) and Z-score (BMD difference in SD in comparison with healthy age- and gender-matched individuals, as provided by the manufacturer). The TBS values were expressed as Z-score based on normal reference ranges derived from the Italian population [27]. Fractured vertebrae were excluded from DXA analyses and data from LS scans were used only if at least three vertebrae were visualized without interfering artifacts. Osteoporosis was diagnosed in the presence of T-score ≤ −2.5 at any site, according to the World Health Organization (WHO) definition, even in subjects before 50 years of age, as suggested by the International Osteoporosis Foundation (IOF) [26,28]. The annual changes in BMD and TBS were expressed as percentage difference and were considered significant when higher than the least significant change (LSC), calculated as 2.8 x coefficient of variation (2.8%, 4.8%, 5.0% and 5.3% for LS, TH, FN scans and TBS, respectively) [29]. Conventional spine radiographs in lateral and anteroposterior projection (T4–L4) were obtained with a standardized technique. Vertebral fractures were diagnosed on visual inspection using the semiquantitative assessment on spine radiographs previosly described by Genant. Vertebrae were assessed as intact (<20% reduction in anterior, middle, or posterior vertebral height) or as having approximately mild (20–25% compression), moderate (25–40% compression) or severe (>40% compression) deformity. Two trained physicians reviewed the radiographs independently and discussed questionable cases to agree on a diagnosis. We calculated the spine deformity index (SDI) by summing for each patient the grade of each vertebra from T4 to L4.

The DXA data were available from all patients at baseline and 24 months after LTx and from 55 patients at last follow-up. TBS data were available from 58 patients at baseline, from 46 patients at 24 months and from 49 patients at last follow-up. Spine radiograph data were available from all patients at each timepoint.

### 2.3. Statistical Analysis

Statistical analysis was performed by SPSS version-24.0 statistical package (SPSS-Inc., Chicago, IL, USA). The normality distribution was checked by the Kolmogorov–Smirnov test. Variables were expressed as mean ± standard deviation if normally distributed and as median (interquartile range) if not normally distributed; the comparison was performed using one-way Student *t*-test or Mann–Whitney U-test, respectively, as appropriate. Categorical variables were compared by χ^2^ test or Fisher exact test, as appropriate. ANOVA for repeated measures was used to compare data between baseline and follow-up. The fracture-free survival probability per year was assessed by the Kaplan–Meier method.

*p* values < 0.05 were considered significant.

## 3. Results

The clinical and biochemical characteristics of patients at baseline are reported in Table 1. As compared with nCF, CF patients had lower age, BMI and GC cumulative dose, whereas the waiting time until LTx and the prevalence of untreated hypogonadism were similar in both groups. Baseline bone workup was performed, on average, 4 ± 7 months before LTx. Overall, the prevalence of osteoporosis before LTX was 48.5%, with no differences between CF and nCF patients, while BMD Z-score at all sites was significantly lower in CF group. TBS and prevalence of FX were comparable between CF and nCF patients. Importantly, only 8.8% of patients had received a bone-active therapy before LTx, the mean calcium intake was insufficient and 86.6% of patients had hypovitaminosis-D (25OHD < 75 nmol/L, 30 ng/mL) [24].

The clinical characteristics of the patients at last available follow-up are reported in Table 2. The mean follow-up duration was 7.3 ± 2.0 (range 5–13) years, with no difference between CF and nCF groups; 33 patients (48.5%) had organ rejection and 17 (25.0%) were still taking a high dose of GC (>5 mg prednisone per day).

**Figure 1:**
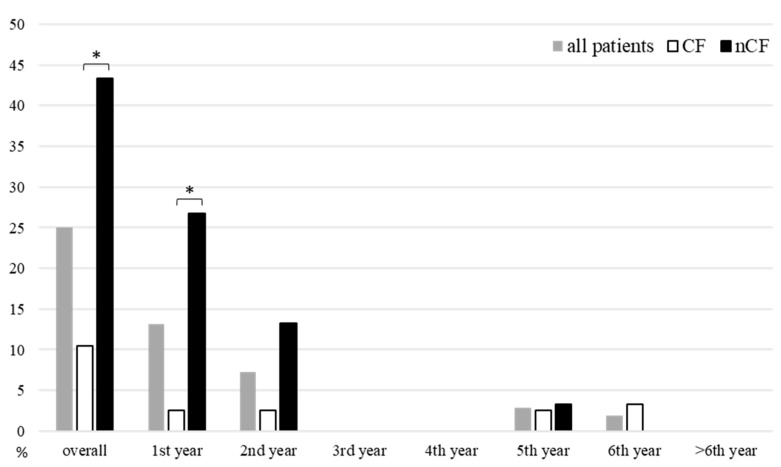
Fracture rate after lung transplantation. CF: cystic fibrosis. nCF: non-CF end-stage lung disease. * *p* < 0.05.

During the whole follow-up, we recorded 17 FX (10 clinical vertebral, 1 morphometric vertebral and 6 nonvertebral) occurring in 13 patients (4 in CF and 13 in nCF patients) with an incidence of 10.5% in CF group and 43.3% in nCF group (*p* = 0.002) (Figure 1). After the first two years of follow-up, the FX rate reduced significantly in the whole cohort (16.2% vs. 4.4%, *p* = 0.045) and in the nCF group (30% vs. 3.3%, *p* = 0.010) and remained stable in CF patients (5.3% vs. 5.3%, *p* = 1.000). As compared with CF patients, patients with nCF had significantly higher FX rate during the first year after LTx but not during the subsequent follow-up period (Figure 1).

Only three patients (4.4%) had incident FX after the second year post-LTx and all fractures were not at spine and fractured patients were as follows: a 28-year-old CF female with hip FX five years after LTx, a 58-year-old CF female with wrist FX six years after LTx and a 65-year-old nCF male with wrist FX five years after LTx. The 28-year-old CF patient and the nCF 65-year-old male patient were still taking pharmacological treatment because of persistent high FX risk due to organ rejection, whereas the 58-year-old CF patient had never taken a bone-active drug during the whole follow-up. The vertebral FX rate markedly decreased from the first two years after LTx to the subsequent period of follow-up (16% vs. 0%, *p* = 0.0006), whereas the rate of nonvertebral FX was not significantly different between the first 2 years of follow-up and the subsequent period (8.8% vs. 4.4%, *p* = 0.490).

**Figure 2:**
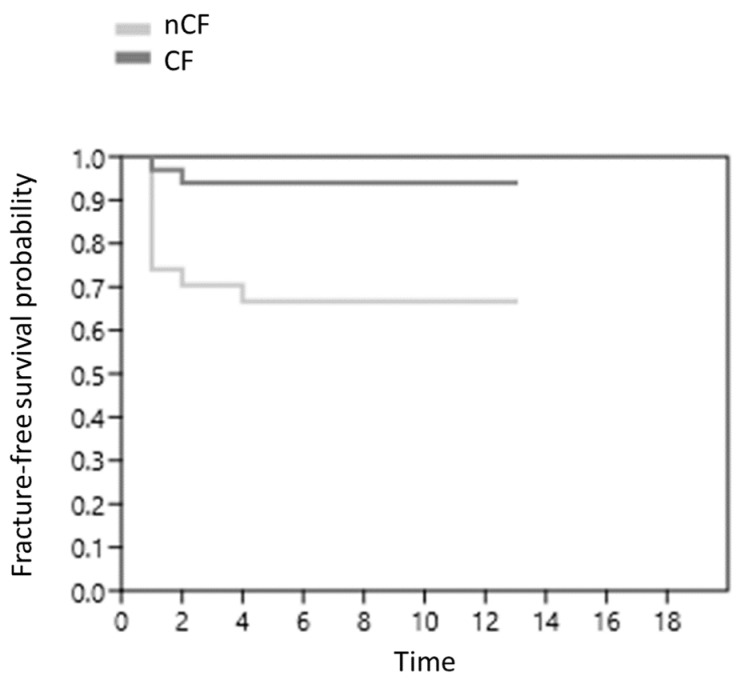
Fracture-free survival probability after lung transplant in patients with cystic fibrosis and other end-stage lung disease. CF: cystic fibrosis. nCF: non-CF end-stage lung disease.

The Fx-free survival probability after LTx in patients with cystic fibrosis and other end-stage lung disease is represented in Figure 2. Overall, the Fx-free survival probability was lower in patients with nCF as compared with CF. According to the logRANK test, this difference was statistically significant (*p* = 0.006). Overall, the HR for FX was 6.9 for nCF patients as compared with CF ones. Stratifying the analysis in early (first 2 years post-LTx) and late (from the third year post-LTx) time points, we calculated that the HR passed from 5.5 *p* = 0.012 to 0 *p* = 1, respectively.

**Figure 3:**
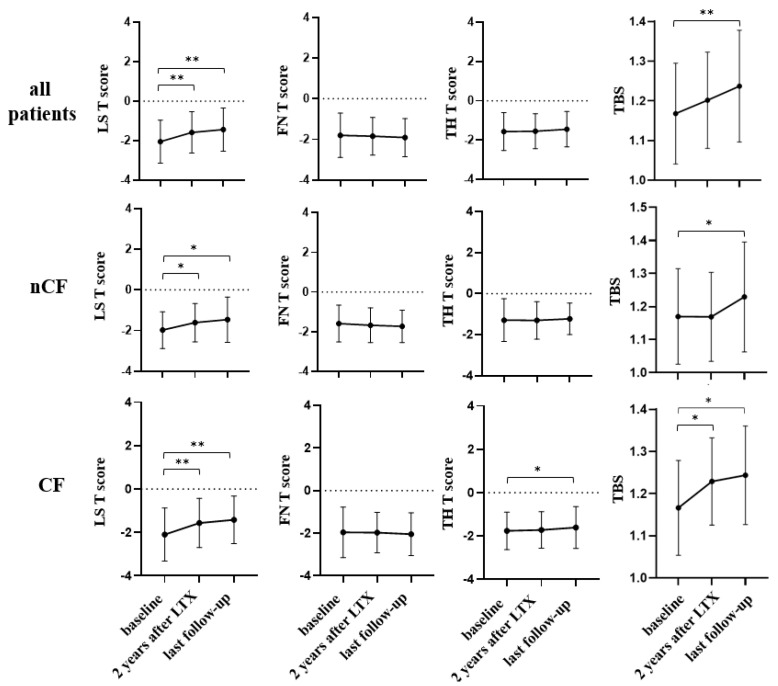
Bone mineral density and trabecular bone score variations after lung transplantation. Data are expressed as mean ± standard deviation. CF: cystic fibrosis. nCF: non-CF end-stage lung disease. LTx: lung transplantation. LS: lumbar spine. FN: femoral neck. TH: total hip. TBS: trabecular bone score. * *p* < 0.05. ** *p* < 0.001.

The BMD and TBS variations after LTx are reported in Figure 3. In both groups, BMD and TBS remained unchanged after the second year post-LTx. In the whole sample, we observed a significant increase in LS T-score and in TBS at the last follow-up available as compared with baseline, but TH T-score improved significantly only in CF patients. In the whole sample, the prevalence of osteoporosis at baseline and at the last follow-up available was similar (48.5% vs. 41.8%, *p* = 0.450).

A total of 17 patients (25%) were taking bone-active therapy after the second year post-LTx, of which 11 (20.4%) were also treated before and 6 (8.8%) introduced a bone-active drug only later. The compliance to bone-active therapy was assessed at each time point and was >85% in all patients. As expected, since the therapy discharge was tailored according to the fracture risk, the patients taking bone-active therapy after the second year post-LTx had a more severe skeletal impairment. They had higher FX rate during the first two years (33.3%) and SDI (4 ± 6), lower LS T-score (−1.6 ± 0.9) and FN T-score (−2.2 ± 0.8) at the second year post-LTx as compared with patients who were not taking bone-active therapy (8.5%, *p* = 0.027, 0.6 ± 1.4, *p* = 0.005, −0.7 ± 0.9, *p* = 0.026, 1.6 ± 0.9 *p* = 0.016, respectively). Nevertheless, after the second year post-LTx, BMD and TBS did not change significantly between patients taking bone-active therapy and untreated patients and there was no difference in the FX rate between treated patients and untreated patients in this time frame.

## 4. Discussion

In our study carried out in a cohort of long-term LTx survivors, we observed that the rate of FX, which is known to be very high in the first two years post-LTx as compared with the general population [30], significantly decreased afterwards. The BMD and TBS remained stable after the second year post-LTx but, as compared with baseline; we observed a significant improvement in bone density and bone quality at LS in both groups, whilst femur BMD ameliorated only in CF patients. After the second year post-LTx the skeletal clinical course was similar between CF and nCF.

Literature data report that solid organ transplant recipients are at enhanced FX risk after surgery and, among others, LTx recipients are the ones at highest risk, with an FX rate between 15–37% [12,14]. Our study shows that the FX risk in LTx recipients is not consistent over time, being significantly higher during the first two years post-LTx, despite bone-active therapy, and markedly decreasing afterwards, despite the withdrawal of bone-active therapy in most patients [12]. The fact that only three subjects had FX after the second year post-LTx, of which one had never been treated with bone-active drugs and two were treated only from the time of LTx onwards, suggests that, in the long term, the occurrence of FX may concern only patients who had not been adequately treated in the peri-transplant period or the frailest ones. Even if the underlying lung disease seems to play a decisive role in the early FX risk after LTx, this may be not the case later on. Indeed, the FX rate was significantly higher in nCF group (30%) than in CF group (5.3%) during the first two years after-LTx but decreased and became similar to that of the CF group thereafter (respectively, 3.3% and 5.3%). This finding is quite different to that obtained in a previous study on a cohort of 210 LTx patients (mostly nCF), in which the authors found a much lower fracture rate, both overall (8%) and in the first year (4.8%), decreasing over time [18]. However, the present study is hardly comparable with the previous one in terms of the percentage of subjects on bone-active therapy (>80% vs. ~50%), of the available follow-up (5 vs. 7 years) and of the type of vertebral fractures (clinical and morphometric in our study vs. only clinical in the previous study). This latter point deserves interest, as the occurrence of morphometric vertebral FX is not negligible in these patients [12]. Regarding the CF group, in our study, the rate of FX post-LTx is constant over time. Even in this subgroup, we found a higher prevalence of post-LTx FX (52.8%) than in the study of Durette et al., reporting a 21% prevalence of FX in a cohort of 86 CF patients [22]. This discordance may be explained considering that, in that study, vertebral morphometry was performed only in half of the patients, and only half of the FX could have been reliably attributed to the post-LTx period of time [22].

As far as BMD is concerned, we observed that, overall, it remained stable after the second year after LTx. The prompt introduction of a bone-active therapy in the vast majority of nCF subjects in the peri-transplant period presumably prevented the femoral BMD loss that usually occurs early after surgery in untreated patients, as previously reported [28]. At variance with the present study, previous data by Durette and colleagues showed that, in CF patients, the BMD at 5–10 years post-LTx may fail to reach the pretransplant values, despite a large use of bone-active therapy both in the peri-transplant period and thereafter [22]. A possible explanation for such a difference could be the timing of baseline workup. Indeed, since in the study by Durette and colleagues the baseline BMD assessment was performed 22 months on average before LTx, a further BMD worsening occurring in the immediate pre-LTx years might not have been detected [22].

In the present study, the bone quality, as estimated by TBS, has been shown to ameliorate over time after LTx both in CF and in nCF group but the trend of TBS was dissimilar in the two groups. Indeed, in this study, we confirmed that, in the CF group, the bone quality amelioration may be precocious, beginning at the moment of the LTx, while it is more delayed, starting from the third year post-LTx in the nCF group. As for other forms of secondary osteoporosis (e.g., glucocorticoid osteoporosis), TBS may explain the discrepancy between the BMD behavior and the FX risk [31]. Indeed, it is conceivable that the difference in bone quality behavior may explain the difference in the vertebral FX rate observed during the first two years in CF and nCF patients, who both showed positive changes in LS BMD without difference between the two groups.

The change in the site of FX over time (i.e., mainly at the vertebral site in the first two years and only at peripheral sites thereafter) can be explained by the fact that vertebrae are rich in trabecular bone, which is very sensitive to GC use, even in the short term [32]. Indeed, during the first two years after LTx, transplanted patients are treated with a very high dose of GC and nCF patients have a longer exposure to GC as compared with CF. Nevertheless, with prolonged exposure to GC, the porosity of cortical bone increases and even skeletal sites rich in cortical bone become more fragile [32]. In keeping with this, the BMD at hip meliorated only in CF patients and only at last follow-up and the rate of peripheral FX did not change over time [12].

This study has some strengths and some limitations. The strength of our study is firstly related to the fact that the baseline assessment was systematically performed for all patients very close to the LTx and the periodical re-evaluation always included the vertebral morphometry. Secondly, information on bone quality, even though by means of an indirect marker such as TBS, has been obtained for the first time in these subjects consenting to show a different behavior of LTx-related bone damage between patients with nCF and those with CF. Finally, this is the first study showing the long-term skeletal outcomes in LTx patients being evaluated and managed appropriately from the moment of LTx and periodically re-evaluated thereafter.

The limitations of the present study are related firstly to the relatively low sample size and secondly to the fact that data were not available for all patients at each time point due to the retrospective nature of this study. Finally, information on physical activity and on the bone turnover parameters that could have been more informative is lacking.

In conclusion, we already knew that LTx recipients were at high risk of FX, especially at vertebral sites during the first two years after LTx, suggesting the need for taking charge of these patients at the time of LTx listing. Importantly, these new data suggest that, although patients with non-CF lung disease are more often affected by FX during the first two years post-LTx as compared with CF ones, the same cannot be said afterwards. Finally, as for other forms of secondary osteoporosis, TBS could be a sensitive tool to monitor and estimate the risk of FX in transplanted patients [31].

## Figures and Tables

**Table 1:** Characteristics of patients awaiting lung transplantation.

	All Patients (68)	CF (38)	nCF (30)	*p*
Age (years)	41.2 (21.5)	33.0 (15.0)	54.5 (17.3)	0.001
BMI (kg/m^2^)	22.1 ± 3.7	20.4 ± 2.4	24.1 ± 4.1	0.001
Sex (Male)	33 (48.5)	20 (52.6)	13 (43.3)	0.474
Waiting time until LTx (months)	8.0 ± 7.0	7.5 ± 6.8	8.4 ± 6.6	0.607
Prevalent fractures	32 (47.1)	17 (44.7)	15 (50.0)	0.807
Mophometric vertebral fractures	28 (42.4)	14 (48.3)	14 (37.8)	0.457
Spine Deformity Index	1.5 (2.0)	0.0 (1.0)	0.0 (2.0)	0.267
Previous GC therapy	30 (44.1)	10 (26.3)	20 (66.7)	0.001
GC cumulative dose (gr prednisone)	4.8 (4.0)	0.0 (0.3)	3.7 (9.1)	0.001
Untreated hypogonadism	21 (33.3)	8 (23.5)	13 (44.8)	0.108
Calcium intake (mg/day)	625 ± 281	673 ± 290	563 ± 261	0.118
TBS	1.186 ± 0.145	1.166 ± 0.112	1.170 ± 0.144	0.915
TBS Z-score	−3.2 ± 2.0	−3.7 ± 2.0	−3.0 ± 2.0	0.189
LS T-score	−2.0 ± 1.2	−2.2 ± 1.2	−1.8 ± 1.1	0.164
LS Z-score	−1.6 ± 1.3	−2.0 ± 1.2	−1.0 ± 1.3	0.006
FN T-score	−1.9 ± 0.9	−2.1 ± 0.9	−1.6 ± 0.9	0.039
FN Z-score	−1.4 ± 1.1	−1.8 ± 1.1	−0.8 ± 0.9	0.001
TH T-score	−1.6 ± 0.9	−1.8 ± 0.8	−1.3 ± 1.0	0.001
TH Z-score	−1.3 ± 1.0	−1.6 ± 0.9	−0.8 ± 1.0	0.026
T-score BMD < −2.5 at ≥1 site	33 (48.5)	22 (57.9)	11 (36.7)	0.094
Z-score BMD < −2.0 at ≥1 site	30 (49.2)	21 (63.6)	9 (32.1)	0.021
25OHD (ng/mL)	20.1 (13.7)	24.1 (10.9)	15.0 (13.0)	0.005
25OHD < 30 ng/mL	58 (86.6)	31 (83.8)	27 (90.0)	0.721
25OHD < 10 ng/mL	13 (19.4)	4 (10.8)	9 (30.0)	0.065
Previous bone-active therapy	6 (8.8)	3 (7.9)	3 (10.0)	1.000

Data are expressed as mean ± standard deviation, median and interquartile range or absolute value and percentage in brackets. CF: cystic-fibrosis. nCF: non-CF end-stage lung disease. BMI: body mass index. LTx: lung transplantation. GC: glucocorticoid. DM: diabetes mellitus. DEXA: dual-energy X-ray absorptiometry. TBS: trabecular bone score. LS: lumbar spine. FN: femoral neck. TH: total hip. BMD: bone mineral density. 25OHD: 25-hydroxyvitamin-D.

**Table 2:** Characteristics of patients at the last follow-up available after lung transplantation.

	All Patients (68)	CF (38)	nCF (30)	*p*
Spine Deformity Index	0 (2)	0 (1)	0.5 (4)	0.114
Prevalent fractures	36 (52.9)	19 (50.0)	17 (56.7)	0.474
GC cumulative dose (gr prednisone)	23.0 (11.0)	21.2 (9.1)	27.0 (19.8)	0.005
High doses of GC at follow-up	17 (25)	9 (23.7)	8 (26.7)	0.786
TBS	1.199 ± 0.205	1.243 ± 0.117	1.229 ± 0.242	0.731
TBS Z-score	−2.4 ± 1.4	−2.5 ± 1.4	−2.1 ± 1.5	0.383
LS T-score	−1.4 ± 1.1	−1.4 ± 1.1	−1.5 ± 1.1	0.795
LS Z-score	−0.9 ± 1.1	−1.1 ± 1.1	−0.5 ± 1.1	0.036
FN T-score	−1.9 ± 0.9	−2.1 ± 1.0	−1.7 ± 0.8	0.195
FN Z-score	−1.2 ± 1.0	−1.6 ± 1.0	−0.7 ± 0.6	0.001
TH T-score	−1.4 ± 0.9	−1.6 ± 1.0	−1.2 ± 0.8	0.149
TH Z-score	−1.0 ± 1.0	−1.4 ± 1.0	−0.6 ± 0.8	0.002
T-score BMD < −2.5 at ≥1 site ^	23 (41.8)	23 (53.1)	6 (23.1)	0.057
Z-score BMD < −2.0 at ≥1 site ^	16 (29.1)	14 (43.8)	2 (8.7)	0.006
Bone-active therapy at transplant	54 (79.4)	28 (73.7)	26 (86.7)	0.236
Bone-active therapy at follow up	17 (25.0)	6 (15.8)	11 (36.7)	0.089
Rejection	33 (48.5)	18 (47.4)	15 (50.0)	1.000

Data are expressed as mean ± standard deviation, median and interquartile range or absolute value and percentage in brackets. CF: cystic fibrosis. nCF: non-CF end-stage lung disease. LTx: lung transplantation. GC: glucocorticoid. TBS: trabecular bone score. LS: lumbar spine. FN: femoral neck. TH: total hip. BMD: bone mineral density. ^ BMD at last follow was available from 55 patients, 23 nCF and 32 CF.

## Data Availability

Not applicable.
